# The Dynamic Coupling of Goal Engagement and Cognition in Daily Life

**DOI:** 10.1093/geroni/igaf122.186

**Published:** 2025-12-31

**Authors:** Jeremy Hamm, Jacob Shane, Jacqueline Mogle, Laura Klepacz, Matthew Pierce, Kelly Parker

**Affiliations:** North Dakota State University, Fargo, North Dakota, United States; Brooklyn College and the Graduate Center, The City University of New York, Brooklyn, New York, New York, United States; RTI Health Solutions, Durham, North Carolina, United States; North Dakota State University, Fargo, North Dakota, United States; North Dakota State University, Fargo, North Dakota, United States; North Dakota State University, Fargo, North Dakota, United States

## Abstract

Consistent evidence shows that investing time and persistent effort toward valued goals (goal engagement) is linked to adaptive development and healthy cognitive aging. However, little is known about the dynamic nature of goal engagement in daily life and the extent to which fluctuations in this motivational resource are linked to day-to-day changes in cognitive functioning. Using 14 days of data from an ongoing measurement-burst study (n = 217, Mage=54±15, 65% female), we examined the extent to which day-to-day changes in goal engagement predicted corresponding shifts in daily episodic memory and executive functioning when controlling for time, age, sex, and education. We assessed daily goal engagement using the persistence in goal engagement scale (e.g., “When I encountered problems today, I didn’t give up until I solved them”), daily episodic memory using a 15-word immediate recall task, and daily executive functioning using a letter fluency task. Multilevel models showed that daily, within-person increases in goal engagement predicted corresponding increases in daily executive functioning (γ=.58, p = .013), but not episodic memory. Between-person differences were also observed, such that middle-aged and older adults with higher goal engagement than their peers had higher average levels of episodic memory performance (γ = .65, p = .016), but not executive functioning. Our findings inform lifespan theories of motivation and development by (a) providing initial evidence that goal engagement exhibits meaningful, within-person fluctuations at the daily level (ICC = .46) and (b) showing that these day-to-day changes are linked to dynamic fluctuations in daily cognition.

